# Correction: Self-assembled fibrillar networks comprised of a naturally-occurring cyclic peptide—LOB3

**DOI:** 10.1039/d0ra90035d

**Published:** 2020-04-07

**Authors:** M. A. Rogers, Q. Feng, V. Ladizhansky, D. B. Good, A. K. Smith, M. Corridini, D. A. S. Grahame, B. C. Bryksa, P. D. Jadhav, S. Sammynaiken, L.-T. Lim, B. Guild, Y. Y. Shim, P.-G. Burnett, M. J. T. Reaney

**Affiliations:** Department of Food Science, University of Guelph Guelph ON N1G 2W1 Canada mroger09@uoguelph.ca +1 519 824 4120 ext. 54327; Department of Physics, University of Guelph Guelph ON N1G 2W1 Canada; Department of Food Science, Rutgers University New Brunswick NJ 08901-8520 USA; Department of Plant Sciences, University of Saskatchewan Saskatoon SK S7N 5A8 Canada; Saskatchewan Structural Sciences Centre, University of Saskatchewan Saskatoon SK S7N 5C9 Canada; Guangdong Saskatchewan Oilseed Joint Laboratory, Department of Food Science and Engineering, Jinan University Guangzhou Guangdong 510632 People’s Republic of China

## Abstract

Correction for ‘Self-assembled fibrillar networks comprised of a naturally-occurring cyclic peptide—LOB3’ by M. A. Rogers *et al.*, *RSC Adv.*, 2016, **6**, 40765–40776.

The authors regret the omission of the following conflict of interest statement.

Dr Martin J. T. Reaney is the founder of, and has an equity interest in, Prairie Tide Diversified Inc. (PTD, Saskatoon, SK, Canada: previous company name is Prairie Tide Chemicals Inc.). Dr Youn Young Shim is a Market Consultant for PTD in Korea. The terms of this arrangement have been reviewed and approved by the University of Saskatchewan in accordance with its conflict of interest policies.

The Royal Society of Chemistry apologises for these errors and any consequent inconvenience to authors and readers.

## Supplementary Material

